# Effective Filtering of Query Results on Updated User Behavioral Profiles in Web Mining

**DOI:** 10.1155/2015/829126

**Published:** 2015-06-10

**Authors:** S. Sadesh, R. C. Suganthe

**Affiliations:** ^1^Velalar College of Engineering and Technology, Thindal, Tamil Nadu, India; ^2^Kongu Engineering College, Perundurai, Tamil Nadu, India

## Abstract

Web with tremendous volume of information retrieves result for user related queries. With the rapid growth of web page recommendation, results retrieved based on data mining techniques did not offer higher performance filtering rate because relationships between user profile and queries were not analyzed in an extensive manner. At the same time, existing user profile based prediction in web data mining is not exhaustive in producing personalized result rate. To improve the query result rate on dynamics of user behavior over time, Hamilton Filtered Regime Switching User Query Probability (HFRS-UQP) framework is proposed. HFRS-UQP framework is split into two processes, where filtering and switching are carried out. The data mining based filtering in our research work uses the Hamilton Filtering framework to filter user result based on personalized information on automatic updated profiles through search engine. Maximized result is fetched, that is, filtered out with respect to user behavior profiles. The switching performs accurate filtering updated profiles using regime switching. The updating in profile change (i.e., switches) regime in HFRS-UQP framework identifies the second- and higher-order association of query result on the updated profiles. Experiment is conducted on factors such as personalized information search retrieval rate, filtering efficiency, and precision ratio.

## 1. Introduction

With the increasing population of web users in Internet and rapid advancement in web communities, effective filtering of query results has been the subject of research work. On one hand, tremendous volume of information provides opportunity for the web user to easily access and share information with each other whereas on the other hand, higher performance filtering rates of results to the end users are not ensured resulting in the problem of information overload to the users and minimize the personalized result rate. Facing such a tremendous volume of information, it becomes a significant challenge to help the web users to improve the query result rate on dynamics of user behavior over time.

To improve the query result rate on dynamics of user behavior over time, we propose the Hamilton Filtered Regime Switching User Query Probability (HFRS-UQP) framework in this paper. The core idea is that by filtering and switching data mining operations, user results are filtered based on personalized information to help web client users to satisfy the web user specific needs. Similar research [1, 2] has been conducted on the search engine logs. However, rapid growth of web page recommendation and need for personalized user profile based prediction of result in web data mining require us to discover a new framework to address the unsolved issues. Therefore, the contributions of our work include the following:

- To improve the query result rate on dynamics of user behavior over time using Hamilton Filtered Regime Switching User Query Probability (HFRS-UQP) scheme.
- To improve personalized information search retrieval rate using Hamilton's filtering framework where maximizing log-likelihood result is filtered with respect to user behavior profiles through search engine.
- To enhance the filtering efficiency based on effective time series using regime switching that switches personalized interest (i.e., updated profiles) for easy accurate filtering of user interests.
- To filter accurate result from web database using data mining operations and satisfy the web user specific needs through search engine personalization using Hamilton Filtered User Query Probability.

The remaining parts of this paper are structured as follows. In Section 2, the related works on user behavioral profiles in web mining and effective filtering are reviewed. We introduce the framework of the Hamilton Filtered Regime Switching User Query Probability (HFRS-UQP) in Section 3. The experiments are conducted in Section 4 and the corresponding results are analyzed in Section 5. Finally, we summarize our concluding work in Section 6.

## 2. Related Works

In this section, we review relevant works in the areas of user behavioral patterns and query filtering results in search logs.

### 2.1. User Behavioral Patterns

User behavioral pattern has been intensively studied to significantly improve the performance of search and query results in the web by incorporating user behavioral profiles for accurate filtering of user results. In [3] uncertainty related to user query was handled by applying reverse nearest neighbor (RNN) that was based on the search made through nearest user with the main aim of improving the results of the query. However, user behavioral aspects remained unaddressed. This user behavioral aspect on the basis of alias naming was introduced in [4] that provided an insight into recall (i.e., improving the search result) and therefore improved rate of retrieval. The improvement in the rate of retrieval therefore resulted in handling dynamics of users over time with the aid of lexical pattern-based approach. However, with the increase in the user query, due to the lack of personalization of queries, it resulted in time complexity. To handle time complexity, personalization techniques were introduced in [5] to measure the user behavioral patterns for recommending users of unseen items in the web, using collaborative and rule based filtering, resulting in the improvement of the recall rate. Though personalization resulted in the improvement of recall rate, search engine personalization was not carried out.

A collaborative approach was presented in [6] using fuzzy logic technique with the purview of improving semantic search engine personalization to derive user patterns and therefore resulted in the improvement of user patterns being derived. Optimization of user behavioral patterns was performed in [7] through Bayesian approach and expectation maximization technique, aiming at improving the accuracy of the query results being generated. Another method was introduced in [8] to improve the information retrieval at different time periods, using ontological web trading agent. Multidimensional Dynamic Time Warping (MDTW) [9] was introduced for providing an insight into multiple classifiers using nearest neighbors, aiming at increasing the accuracy of query results being generated. A new algorithm for semantic web matching was introduced in [10] to improve the efficiency of web service discovery using service matching algorithm. The characteristics of time in rule mining process were introduced in [11] using Apriori and FP-growth algorithms with the objective of increasing the number of rules generated using temporal rule mining. In [12], browsing of user on the basis of time (i.e., time series analysis) was taken into consideration and based on the results obtained through it, optimization technique called chaos, and swarm was applied and handled the dynamics of user behavior over time.

### 2.2. Query Filtering Results

In this subsection, we mainly focus on one mainstream of web mining applications: query filtering results. Previous research works on user behavioral patterns can be mainly split into two parts. One is trying to identify the query filtering results of similar user to improve the search result rate in web. In [13] Unsupervised Duplicate Detection (UDD) was handled to perform record matching over multiple web databases based on different user results evolved over time. Though information obtained from multiple web databases improved rate of accuracy, but compromised personalization resulting in time complexity. A personalized ontology model was designed in [14] with the main aim of increasing the gathering of web information using multidimensional ontology mining for effective query result. Though query result produced improved with scalable users, quality of web information was compromised. To improve the quality of web of information obtained in web, GA-based evolutionary approach was introduced in [15], improving the quality of service at different time period and attained optimized service composition. Another method for optimized forecasting of query result was introduced in [16] called TAg cloud-based Clinical Trial Search (eTACTS), aiming at minimizing the information overload during gathering of web information, and efficient filtering of search result space. Though effective filtering was achieved, time series analysis for user query was not considered.

In [17], a method with the main aim of increasing the search result delivery was introduced to help the web users in identifying the required contents in web using graph based approach. Dynamics of user behavior over time for other users were also provided through these graph based approaches, improving the search engine personalization, but lacked query optimization. In order to improve the query optimization, in [18], an adaptive cuckoo search method was introduced with the aim of reducing the execution time with the aid of Resource Description Framework (RDF). By applying RDF, personalized interest of user was considered, but scalability remained unsolved. Effective query distribution using search engine on the basis of user query at varied time period was performed in [19] with the objective of purifying the log data population using Top Level Domain (TLD) server. However, filtering results obtained through TLD server included redundant user query, compromising computational complexity. In order to obtain top-k query filtering results and reduce the computational complexity, in [20], a candidate filtering method was applied that enormously improved the whole class of query processing.

Optimization of query distribution was also ensured through efficient caching policy. Query processing for big data was handled in an efficient manner in [21] using E2E model with the aid of sample training strategy. But, forecasting was not handled in an efficient manner. In [22], web site visit forecasting was performed using linear regression algorithms, aiming at producing accurate results and also reduced server maintenance cost. A method was presented in [23] to forecast the navigating pattern of user through two-tier architecture, improving the rate of accuracy of user intuition.

Based on the aforementioned methods and techniques, in this paper, an efficient framework called, Hamilton Filtered Regime Switching User Query Probability (HFRS-UQP), is presented for effective filtering of query results. The elaborate description of this is provided in the forthcoming sections.

## 3. Methodology

In this section, we will introduce and discuss the proposed Hamilton Filtered Regime Switching User Query Probability (HFRS-UQP) framework for effective filtering of query results. To start with, the formulation of research problem is included so that the clear picture of the methodology is given. Then, the framework is further divided into two subprocesses, which are filtering based on user query and regime switching procedure.

### 3.1. Problem Formulation

Intuitively, the effective filtering of query results is to filter user result based on the personalized information on automatic updated profiles through search engine and switch the personalized interest of the web clients. Specifically, the research problem of effective filtering of query results can be formulated as a mapping function *θ* as follows:

(1) $$\begin{array}{c} \alpha =WC*Req*WP. \end{array}$$

In (1) WC represent the web clients, Req denotes the requests made by several web clients to the browser, and WP is the recommending web pages. The foremost objective of the function goal of function *α* is to map the above three entities for query result rate on dynamics of user behavior over time. In the forthcoming two subsections, we will detail how to filter based on user query and design a regime switching procedure.

**Figure 1:**
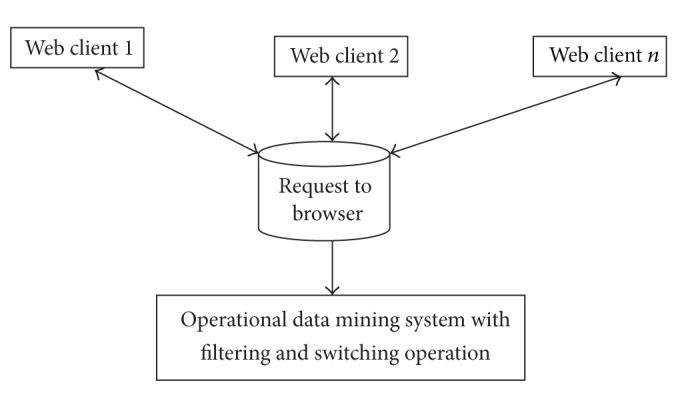
End-to-end query result fetching using data mining operations.

The recommended web pages are mined using data mining operations to access web logs and provide accurate results to the user query. Usage mining allows the user to fetch accurate result with effective filtering method. In our proposed work, data mining operations discover the patterns with accurate result rate. Initial process is to submit the clients query on the search engine. The search engine then sends that query to the database for fetching accurate results. The filtering operation is carried out here to filter out the unfit query result for easy process. The query result fetching through data mining operations is briefly shown in Figure 1.

**Figure 2:**
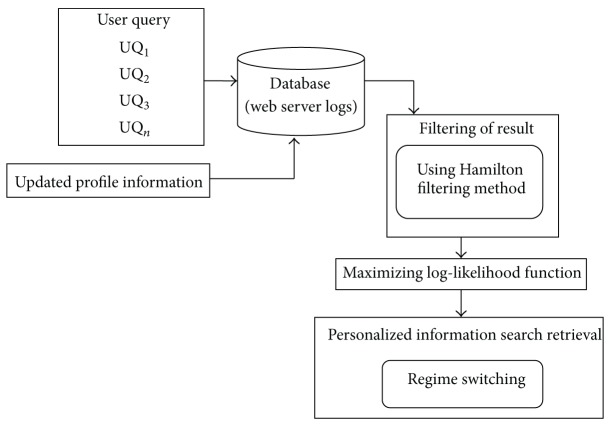
Architecture diagram of HFRS-UQP framework.

Figure 1 shows the end-to-end query result fetching for several web clients in the range of WC_1_, WC_2_,…, WC_*n*_, where their requests are placed on the web browser. The web browser using operational data mining system and data mining operations with effective filtering and switching operations improve the query result rate. The network based mining of query results fetches more accurate results to the users through the web browser. Web mining fetches accurate results for different types of user queries. The architecture diagram of HFRS-UQP framework is shown in Figure 2.

Our proposed framework fetches the user query and performs data mining operations. Web mining extracts the useful information from the server logs and finds out accurate results after the filtering operation. The updated user profiles are also used for the effective filtering of the query result based on their profiles. The query related result with the maximum likelihood function is filtered out by avoiding the unrelated result. Hamilton based filtering method is employed for removing the unrelated object terms from the database. The personalized information is retrieved based on the updated profile using the regime switching.

Search engines utilize a mixture of data mining operations as a means to provide results in a timely manner without compromising the quality rate of results to the users. In HFRS-UQP framework, people search the information on the web and fetch accurate results only when understandable query is submitted on the search engine. Dynamics of user behavior over time system is effective in static and dynamic changes based profile information to fetch the accurate query results.

### 3.2. Filtering Based on User Query

Web usage mining aims to confine the proposed framework once the filtering of results based on user behavioral profiles is performed. Data stored in the usage logs (i.e., database) is used in HFRS-UQP framework for providing solution to quality web query search. The proposed framework enhances the performance by analyzing the submitted query using the sequential distributed form. The sequential distribution form in HFRS-UQP framework orders the query submitted by different users. Each user may submit the same query once, twice, or more than one time resulting in space complexity. The HFRS-UQP framework overcomes this difficulty by introducing the sequential distribution form.

The filtering based on user query takes both the static and dynamic profile information. The dynamic portion of the user information is managed by restoring the policy with intercept variations. The updated inference is used as an input for filtering data mining operation on user query. The entire profile information is used for processing of user query on dynamics of user behavior over time.

#### 3.2.1. Hamilton Filtering Method

Let us consider a sample state user query form “*S*” taken for identifying the filtering rate using data mining operations. A simple framework is constructed for filtering the query result for the submitted user query. The result retrieval system hosts the result with lesser response time. The conditional density of filtering is formularized as

(2) $$\begin{array}{c} S\left(\;UQ\;|\;D_{t}\;\right)=UQ\left[\;D_{x},D_{x+1},D_{x+2},D_{x+3},\ldots ,D_{x+n}\;\right]. \end{array}$$

In (2) the sample “*S*”, user query “UQ” is submitted on the database at time “*t*.” The database contains different result set for the query. Then the different result sets are represented as *D*_*x*_, *D*_*x*+1_, up to *D*_*x*+*n*_. The filtering of the result is based on the user profiles in HFRS-UQP framework. Web searching with filtering framework helps to easily identify the convenient way of fetching the query result. The user query probability is also analyzed and work extended on filtering the sample query result as

(3) $$\begin{array}{c} \text{Filtering of “}S\text{” result}=UQ\left[\;D_{x},D_{x+2}\;\right]. \end{array}$$

**Figure 3:**
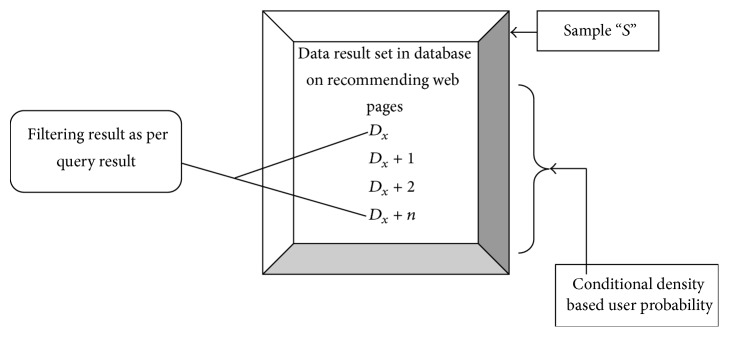
Diagrammatic form of Hamilton's filtering framework.

The user query result fetched on “*S*” after filtering in HFRS-UQP framework satisfies only the *D*_*x*_, *D*_*x*+2_ result set as provided in (3). So the user query probability is fetched using Hamilton's filter. Conditional density based Hamilton's filtering improves the filtering efficiency of web query results. Hamilton's filtering framework is shown in Figure 3.

Figure 3 shows the diagrammatic form of Hamilton's filtering framework. Hamilton's filtering framework solves different case scenarios with diverse period interference. Conditional density based user probability is employed for effective filtering of user query result on web using data mining operations. The dynamic updating of user profiles is also utilized on filtering the results. Clustering is performed on the dynamic profile based on the attribute list. The static user profiles are not clustered and they are placed simply in the separate row of the web server. The filtering using the user profiles (i.e., static and dynamic) fetches accurate result in HFRS-UQP framework based on the maximum log-likelihood function. Maximum log-likelihood function is briefly explained in Section 3.2.2.

#### 3.2.2. Maximize Log-Likelihood Function

Log-likelihood function on the proposed framework is utilized to establish the user query probability result with maximum accurate result rate. The continuous probability log-likelihood distribution function for *P*[*D*_*x*_] and *P*[*D*_*x*+2_] in HFRS-UQP framework is formularized as

(4) $$\begin{array}{c} UQ\left[\;D_{x},D_{x+2}\;\right]=\text{Maximum }P\left[\;D_{x}\;\right]P\left[\;D_{x+2}\;\right]. \end{array}$$

From (4), the query with the accurate result set is fetched for sample “*S*” using maximum likelihood of result based on static and dynamic user profiles. The maximum accurate result is fetched through the log-likelihood function provided in (5). The maximize log-likelihood increasing function applied on web mining fetch accurate query result for users with minimal processing time. Log-likelihood function is formularized as

(5) $$\begin{array}{c} \text{Log-likelihood}=\frac{\partial \log P\left[D\;|\;x,x+2\right]}{\partial P}. \end{array}$$

Log-likelihood achieves the outcome with maximization procedure. The result permits allocation using conditional user probability. The conditional way uses the user profile information to fetch high accurate query result on web. “*x*” and series “*x* + 2” are set of results stored on the web server database to fetch more accurate result.

### 3.3. Regime Switching Procedure

Whenever a query is submitted, the HFRS-UQP framework checks the user profile information status (i.e., static or dynamic) changes. The dynamically updated profile is viewed and information is used for further switching of result as per the individual users need. The system achieves the final web page result with higher level of user satisfaction. Regime switching procedure is shown in a step by step process. In Algorithm 1, the algorithmic step for filtered probability of results is presented. HFRS-UQP framework performs effective regime (i.e., updating) for different set of client profiles for different set of period time “*t*,” where the profiles are updated on the web server. The profiles are then used for possible set of the result state for each user query “UQ.”

#### 3.3.1. Smoothed Probability Switching Procedure

The state estimated for the model of switching is carried out using smoothing probabilities. The smoothing probability in HFRS-UQP framework is formularized as

(6) Smoothed Probability=PD=j ∣ Filtering of  “S”  result.

The smoothed probability “*j*” of database server “*D*” estimated the procedure with accurate result. The filtering of query result is performed effectively with smoothed regime probability “*P*.” The smoothed probability based updating switches to every user updating with higher precision ratio. HFRS-UQP framework with smoothed probability maintains the distinct time series to identify the second- and higher-order association of query result.

## 4. Experiment

In this section, we conducted the experiment in JAVA platform with Amazon EC2 database to evaluate the performance of the proposed Hamilton Filtered Regime Switching User Query Probability (HFRS-UQP) framework in web data mining to retrieve the individual user interest based result.

### 4.1. Database

The main reason for selecting the Freebase Data Dump is that they are in different domains analyzed to identify the efficiency level so that we can examine the performance of the proposed framework in web mining. To evaluate the proposed framework, Freebase Data Dump is used with the updated user profiles to perform the experimental operation. A data dump is the essential information provided on identifying the facts concerning each subject in Freebase. Freebase is an open database of the world's information that covers millions 15 of groups in hundreds of group and our framework is used to fetch the accurate result to the users. It simultaneously contains prearranged information on several popular topics, including 16 movies, music, people and locations. The information includes the historic events, European railway stations, and chemical properties of common food ingredients. The information is supplemented by the efforts of total community of users to identify the accurate result. The user query is run together by using the updated profile information to add the structured information. Present research work is compared against the existing web page recommendation through semantic enhancement method (WR-SE) and concept-based user profiling (CUP) method.

### 4.2. Metrics

Three widely adopted metrics are used in the experiments which include personalized information search retrieval rate, running time, and precision ratio. The personalized information search retrieval rate refers to the accuracy of user request retrieval which is given as follows. It is measured in terms of percentage (%):

(7) $$\begin{array}{c} PISR=\overset{n}{\underset{i=1}{\sum }}\frac{{UR}_{i}}{n}. \end{array}$$

In (7), UR_*i*_ is the user request made and *n* is the number of total requests made in the testing set. The running time is the time taken to perform user request retrieval with the help of conditional density of filtering is given as below. It is measured in terms of milliseconds:

(8) $$\begin{array}{c} R_{time}=Time\left(\;S\left(\;UQ\;|\;D_{t}\;\right)\;\right). \end{array}$$

Precision is the ratio of the number of relevant results retrieved to the total number of irrelevant and relevant results retrieved for the user requests being made. It is usually expressed as a percentage. It is given as

(9) $$\begin{array}{c} P=\frac{{UR}_{R}}{{UR}_{R}+{UR}_{I}}. \end{array}$$

## 5. Results and Discussion

The performance of Hamilton Filtered Regime Switching User Query Probability (HFRS-UQP) framework in web mining is compared with the existing web page recommendation through semantic enhancement method (WR-SE) and concept-based user profiling (CUP) method in web mining. The performance is evaluated according to the following metrics.

### 5.1. Impact of Personalized Information Search Retrieval Rate

**Figure 4:**
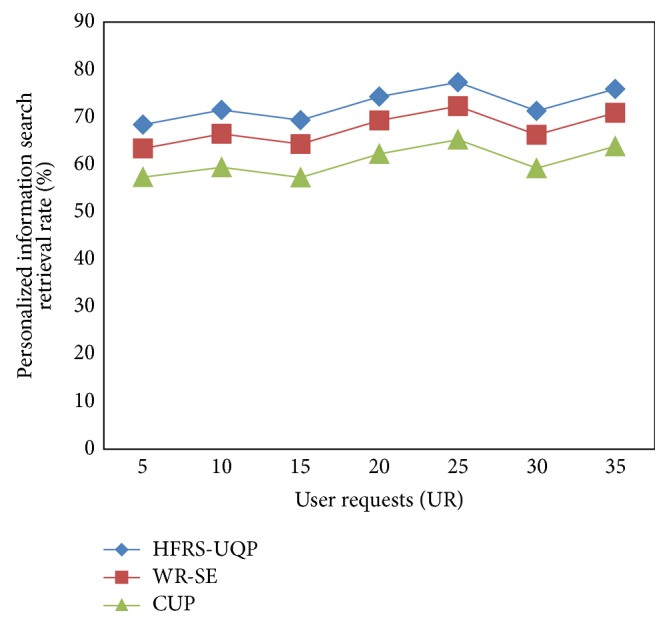
Performance of personalized information search retrieval rate.

Figure 4 shows the result of personalized information search retrieval rate versus the varying number of user requests based on the user requests made regarding different movies provided in Freebase Data Dump. To better perceive the efficacy of the proposed HFRS-UQP framework, substantial experimental results are illustrated in Figure 4 and compared against the existing WR-SE [1] and CUP [2], respectively.

Results are presented for different number of user requests that cover millions of movies related to different theme in hundreds of group in mobile web mining. The personalized information search retrieval rates in web mining for several user requests are performed at and different time interval is shown below. The higher the number of user requests being sent, the more successful the method. The results reported here confirm that, with the increase in the number of user requests being sent to the log files, the search retrieval rate also increases. The process is repeated for 35 user requests for conducting experiments.

In order to investigate the personalized information search retrieval rate required by user requests to perform validations, we implemented verifying both WR-SE and CUP for different implementation runs. As illustrated in Figure 4, the proposed HFRS-UQP framework performs relatively well when compared to two other methods WR-SE [1] and CUP [2]. The personalized information search retrieval rate using HFRS-UQP framework is improved with the application of Hamilton's filtering framework where maximize log-likelihood result is fetched in web mining.

The personalized information search retrieval rate in HFRS-UQP framework is increased by establishing a filtering scheme that helps to easily identify the convenient way of fetching the query result. As a result, the search retrieval rate using HFRS-UQP framework is improved by 6–8% compared to WR-SE. Moreover, the query related result with maximum likelihood function is filtered out by avoiding the unrelated result resulting in the improvement of search retrieval rate using HFRS-UQP framework by 15–17% compared to CUP.

### 5.2. Impact of Running Time

**Figure 5:**
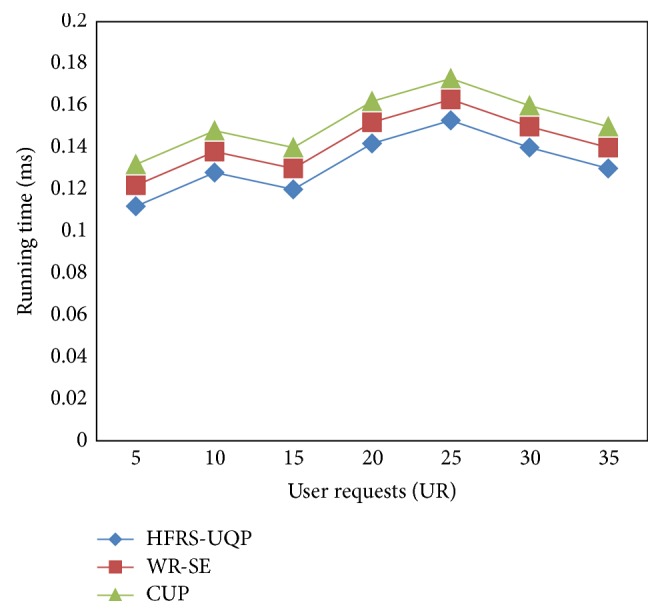
Performance of running time.

In order to reduce the running time with the static and dynamic changes based profile information, the user requests and user profile information for effective handling of both static and dynamic changes is considered according to the user requests regarding different music extracted from Freebase Data Dump. In the experimental setup, the number of user requests ranges from 5 to 35 as illustrated in Figure 5. The running time using the framework method HFRS-UQP provides comparable values more than the state-of-the-art methods.

The targeting results of end-to-end query for measuring running time using HFRS-UQP framework compared with two state-of-the-art methods WR-SE and CUP in Figure 5 is presented for visual comparison based on the number of user requests being sent for extracting different music files. Our framework HFRS-UQP differs from the WR-SE [1] and CUP [2] in that we have incorporated end-to-end query results that employ conditional density of filtering in web mining. With the objective of reducing the running time in HFRS-UQP, different result set for the query is based on the maximum log-likelihood function.

The weight of the user query probability result with maximum accurate result rate is compared and evaluated which helps in the minimization of execution time by 6–8% compared to WR-SE. Furthermore, with the effective application of Hamilton Filtered User Query Probability, the web user specific needs are satisfied at minimum time through search engine personalization by 13–17% compared to CUP.

### 5.3. Impact of Precision Ratio

**Figure 6:**
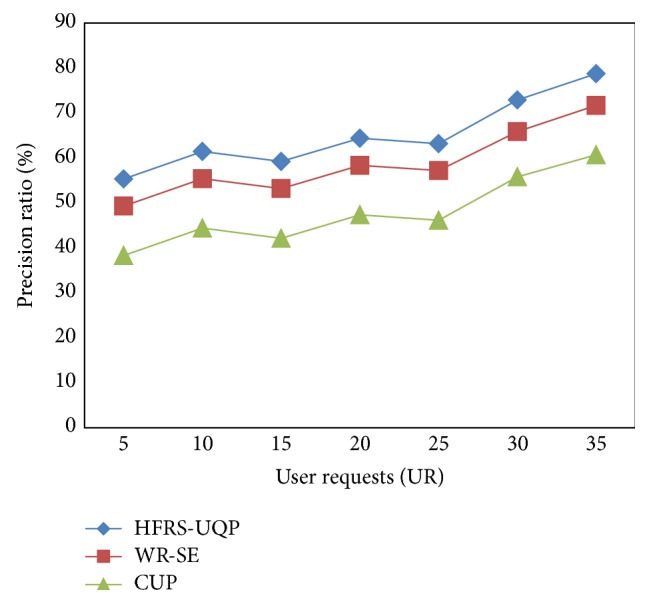
Performance of precision ratio.

Figure 6 shows the precision ratio for HFRS-UQP framework, WR-SE [1] and CUP [2] versus seven different user requests with respect to the location of the origin of music and movies. The precision ratio returned over HFRS-UQP framework increases gradually though not linear for differing user requests in web mining because of the dynamic changes observed.

From Figure 6, it is illustrative that the precision ratio is improved using the proposed framework HFRS-UQP. For example, with 15 user requests, the precision ratio was 59.22 percent using HFRS-UQP whereas WR-SE recorded 53.19 percent and 41.16 percent in CUP. By observing the dense user requests in web mining, the precision ratio is improved. This is because with the application of regime switching scheme, the precision ratio is increased. With the help of regime switching, personalized interest or the updated profiles are switched for easy and accurate filtering of user interests, improving the precision ratio by 8–10% compared to WR-SE. In addition, by applying smoothed probability switching procedure, maintains distinct time series for efficient identification of second and higher order association of query result and therefore improves the precision ratio by 22–30% compared to CUP.

## 6. Conclusion

A Hamilton Filtered Regime Switching User Query Probability (HFRS-UQP) framework to improve the query result rate on dynamics of user behavior over time in web mining is introduced. We then showed how this framework can be extended to incorporate Hamilton's filtering framework to improve the query result rate. The query result rate was improved for both static and dynamic changes based profile information and fetched the accurate query results. These accurate results were arrived using conditional density of Hamilton's filtering framework with the aid of extensive Maximize Log-Likelihood that in turn increases the personalized information search retrieval rate. Next, the maximizing log-likelihood function filters user behavior profiles to improve the precision ratio. Finally, the regime switching effectively switches personalized interest for easy and accurate filtering of user interests. In our experimental results the HFRS-UQP framework with smoothed probability switching procedure showed better performance compared to the state-of-the-art-method over the parameters, personalized information search retrieval rate, running time, and precision ratio. For the future work, a key information extraction method will be developed to compare with the query results on updated user behavioral profiles and an extensive comparison with the existing Hamilton Filtered Regime Switching User Query Probability (HFRS-UQP) framework can be provided.

## Figures and Tables

**Algorithm 1:**
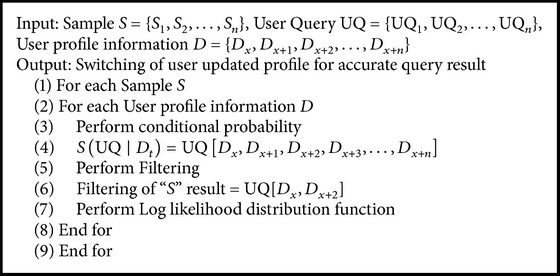
Algorithm of regime switching.
